# Supplementary material for: Functional Connectivity, Tissue Microstructure, and T2 at 11.1 Tesla Distinguishes Neuroadaptive Differences in Two Traumatic Brain Injury Models in Rats: A Translational Outcomes Project in NeuroTrauma (TOP-NT) UG3 Phase Study
Source: Neurotrauma Rep. 2025 Sep 17;6(1):885–902. doi: 10.1177/2689288X251380144 (PMC12543431; doi:10.1177/2689288X251380144)
Supplement: Supplementary Figure S1 [file 2689288x251380144_suppl_figures1.docx]

**
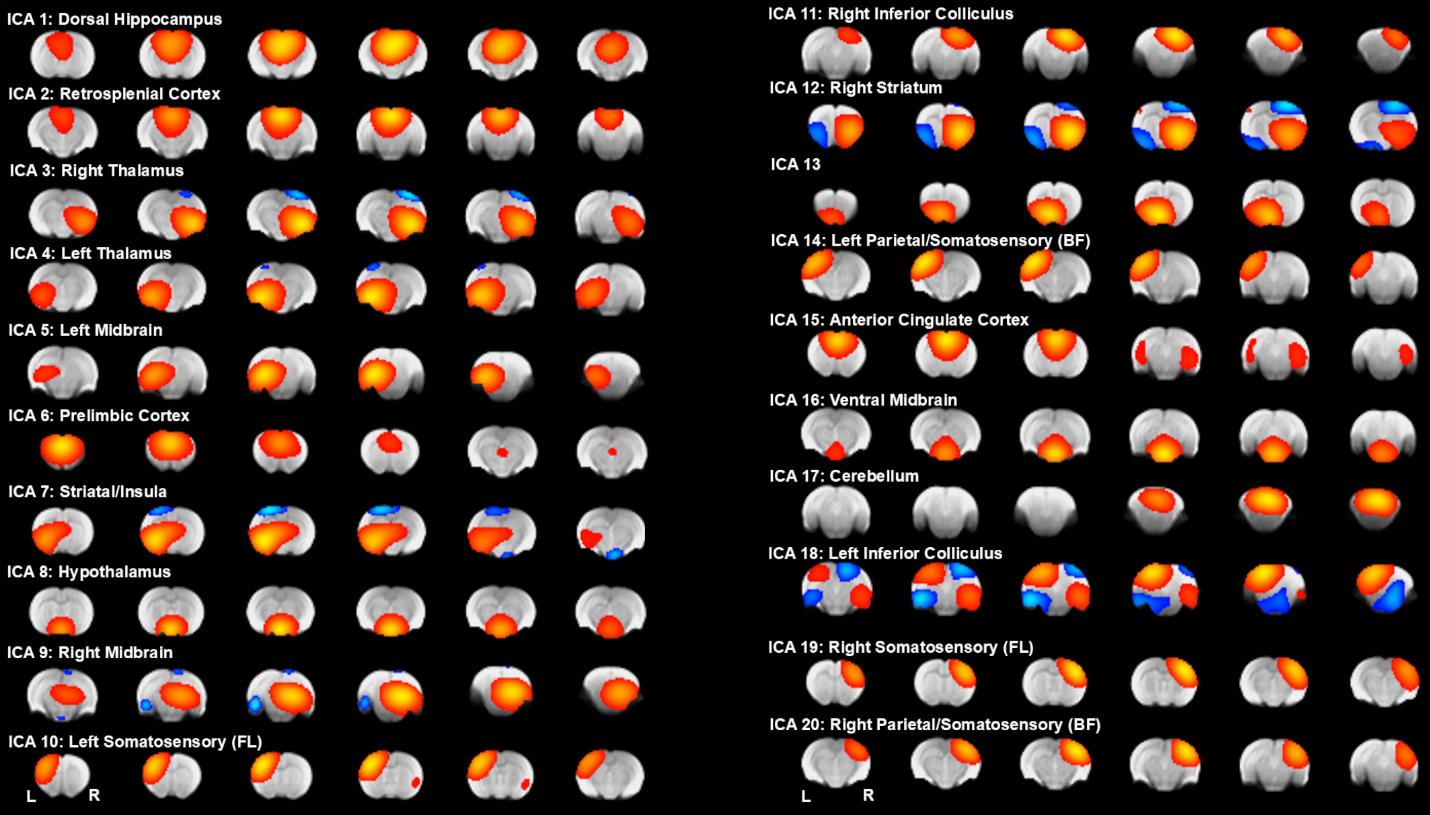
**

**Supplemental Figure 1.** **Group probabilistic independent components analysis of rat resting state functional magnetic resonance images collected on an 11.1 Tesla MRI scanner.** Twenty components were identified across 92 fMRI datasets. Components were classified according to location of peak Z statistic voxel.
