# Supplementary material for: Functional Connectivity, Tissue Microstructure, and T2 at 11.1 Tesla Distinguishes Neuroadaptive Differences in Two Traumatic Brain Injury Models in Rats: A Translational Outcomes Project in NeuroTrauma (TOP-NT) UG3 Phase Study
Source: Neurotrauma Rep. 2025 Sep 17;6(1):885–902. doi: 10.1177/2689288X251380144 (PMC12543431; doi:10.1177/2689288X251380144)
Supplement: Supplementary Figure S2 [file 2689288x251380144_suppl_figures2.docx]

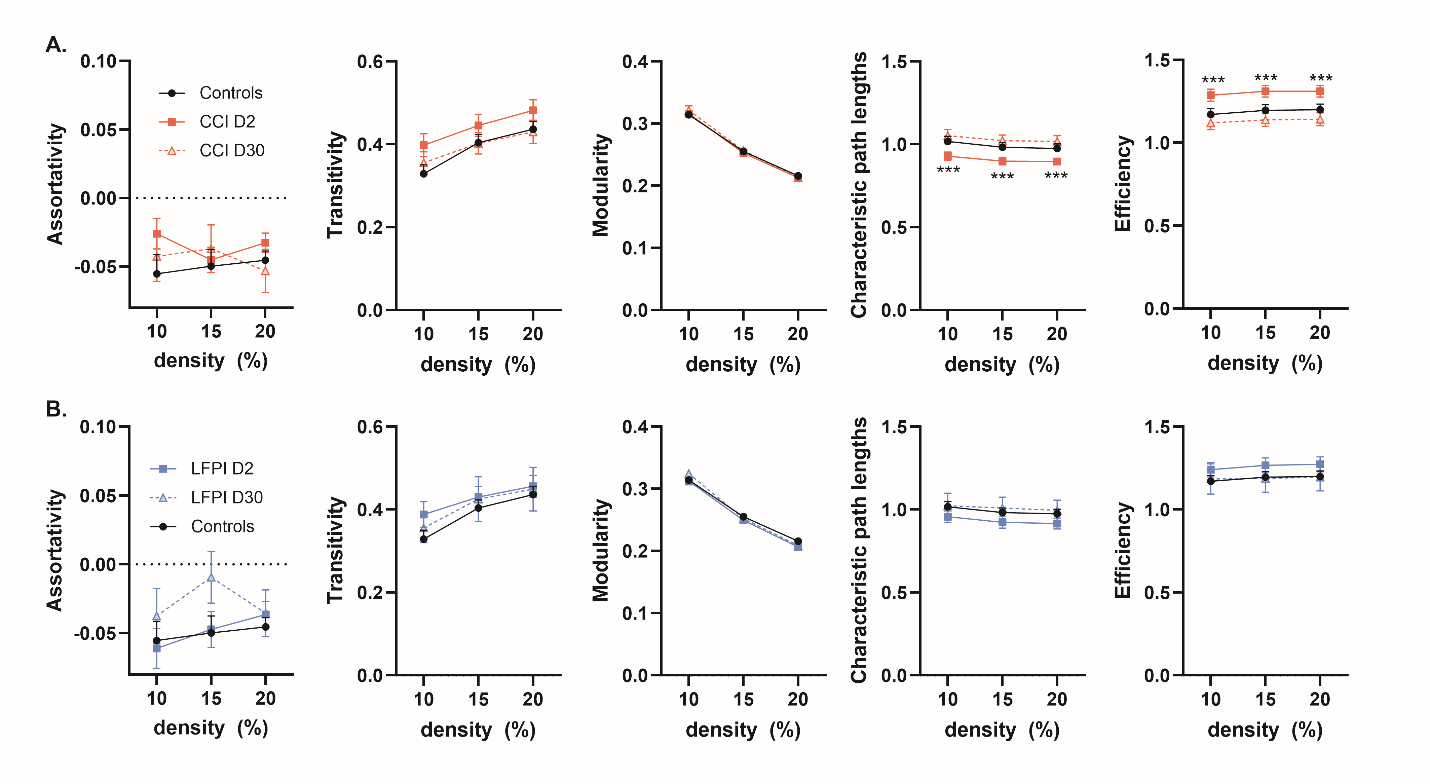


**Supplemental Figure 2.** Estimation of network metrics on randomized versions of matrices shown in Figure 2C-D. Network measures for CCI days 2, 30 and controls calculated at graph density thresholds of 10, 15, and 20%. D) Network measures for LFPI days 2, 30 and controls. Two-way ANOVA comparisons followed by Tukey’s multiple comparisons test (*day 2 vs control, ** day 30 vs control, *** day 30 vs day 2). Data shown as mean ± standard error. The approximate craniotomy/impact site for CCI and LFPI are shown on 3D connectome maps.
